# Metformin Prevents and Reverses Inflammation in a Non-Diabetic Mouse Model of Nonalcoholic Steatohepatitis

**DOI:** 10.1371/journal.pone.0043056

**Published:** 2012-09-18

**Authors:** Yuki Kita, Toshinari Takamura, Hirofumi Misu, Tsuguhito Ota, Seiichiro Kurita, Yumie Takeshita, Masafumi Uno, Naoto Matsuzawa-Nagata, Ken-ichiro Kato, Hitoshi Ando, Akio Fujimura, Koji Hayashi, Toru Kimura, Yinhua Ni, Toshiki Otoda, Ken-ichi Miyamoto, Yoh Zen, Yasuni Nakanuma, Shuichi Kaneko

**Affiliations:** 1 Department of Disease Control and Homeostasis, Kanazawa University Graduate School of Medical Science, Ishikawa, Japan; 2 Department of Hospital Pharmacy, Kanazawa University Graduate School of Medical Science, Ishikawa, Japan; 3 Division of Clinical Pharmacology, Department of Pharmacology, School of Medicine, Jichi Medical University, Tochigi, Japan; 4 Genomic Science Laboratories, Dainippon Sumitomo Pharma Co. Ltd., Osaka, Japan; 5 Department of Human Pathology, Kanazawa University Graduate School of Medical Science, Ishikawa, Japan; Centro de Investigación en Medicina Aplicada (CIMA), Spain

## Abstract

**Background:**

Optimal treatment for nonalcoholic steatohepatitis (NASH) has not yet been established, particularly for individuals without diabetes. We examined the effects of metformin, commonly used to treat patients with type 2 diabetes, on liver pathology in a non-diabetic NASH mouse model.

**Methodology/Principal Findings:**

Eight-week-old C57BL/6 mice were fed a methionine- and choline-deficient plus high fat (MCD+HF) diet with or without 0.1% metformin for 8 weeks. Co-administration of metformin significantly decreased fasting plasma glucose levels, but did not affect glucose tolerance or peripheral insulin sensitivity. Metformin ameliorated MCD+HF diet-induced hepatic steatosis, inflammation, and fibrosis. Furthermore, metformin significantly reversed hepatic steatosis and inflammation when administered after the development of experimental NASH.

**Conclusions/Significance:**

These histological changes were accompanied by reduced hepatic triglyceride content, suppressed hepatic stellate cell activation, and the downregulation of genes involved in fatty acid metabolism, inflammation, and fibrogenesis. Metformin prevented and reversed steatosis and inflammation of NASH in an experimental non-diabetic model without affecting peripheral insulin resistance.

## Introduction

Nonalcoholic steatohepatitis (NASH) refers to a stage within the spectrum of nonalcoholic fatty liver disease (NAFLD) characterized by hepatic steatosis, inflammation, and fibrosis, and is emerging as one of the most common liver diseases and a leading cause of cryptogenic cirrhosis [1].

While searching for clinical factors predicting outcomes from liver fibrosis, a key feature associated with the progression of cirrhosis and hepatocellular carcinoma, we found that tight glycemic control by diet or bolus-first insulin therapy ameliorated liver fibrosis [2]. Indeed, diabetes is an independent risk for the progression of liver fibrosis in hepatitis C [3]. Experimentally, diabetes accelerates the pathology of steatohepatitis in the type 2 diabetic rat model OLETF fed a methionine and choline-deficient diet [3]. These findings suggest that a diabetic state itself is an added risk for liver fibrosis. From this perspective, the insulin sensitizing anti-diabetic agent pioglitazone ameliorates NASH pathology in patients with type 2 diabetes [4]. However, the effects of pioglitazone on liver pathology seem only marginal in non-diabetic patients with NASH [5]. Furthernore, both vitamin E and pioglitazone failed to improve hepatic fibrosis in non-diabetic patients with NASH [6]. This study again stated that additional anti-fibrogenic therapy should be required in non-diabetic patients with NASH.

The anti-diabetic drug metformin restrains hepatic gluconeogenesis through pleiotropic effects including activation of AMP-activated protein kinase (AMPK) [7], suppression of glucose-6-phosphatase expression [8], and inhibition of mitochondrial oxidative phosphorylation [9], which may play a pivotal role in glucose and lipid metabolism in the liver [10], [11]. We previously performed a DNA microarray analysis on the livers of obese diabetic db/db mice 2 h after a single administration of metformin and showed that metformin altered the expression level of multiple genes linked to glucose and lipid metabolism in the liver [12]. *In vitro* studies suggest that AMPK suppresses proliferation and activation of hepatic stellate cells by inhibiting Akt, inducing antioxidant enzymes, and blocking the cell cycle [13], [14].

However, evidence for the use of metformin in the treatment of NAFLD is still limited to hepatic steatosis [15]–[17], and the effects of metformin on hepatic inflammation and fibrosis, key histological features of NASH, remains unclear. In the present study, we assessed whether metformin ameliorated and/or reversed inflammation and fibrosis in an experimental NASH mouse model without diabetes.

## Materials and Methods

### Ethics Statement

The animal study was carried out in accordance with the Guidelines on the Care and Use of Laboratory Animals issued by Kanazawa University. The protocol was approved by the ethical committee of Kanazawa University (Approval NO. 070816). All surgery was performed under sodium pentobarbital anesthesia, and all efforts were made to minimize suffering.

### Animal model and experimental design

Eight-week-old C57BL/6 mice were obtained and housed in a room under controlled temperature (25°C), humidity, and lighting (12/12-h artificial light/dark cycle). Animals were given free access to standard laboratory rat chow and tap water. C57BL/6 mice were divided into three experimental groups and fed for 8 weeks as follows: a) normal chow (NC, *n* = 10), b) methionine- and choline-deficient diet (MCD)+high fat (HF) diet (Oriental Yeast Co., Tokyo, Japan; MCD, fat 60%, *n* = 20), c) MCD+HF diet mixed with 0.1% metformin (MCD+Met, fat 60%, *n* = 25). In our previous study [3], we established a dietary rodent model of steatohepatisis associated with insulin resistance by feeding a methionine choline-deficient and high fat diet (MCD+HF). This model reveals only mild inflammation with no fibrosis at 4 weeks and develops intense lobular inflammation and perivenular and pericellular fibrosis prominently in liver at 8 weeks. Based on these time course findings, we fed MCD+HF for 8 weeks to create the advanced stages of steatohepatitis. We set the dose if metformin at 37.5 mg/kg of mouse weight, corresponding to 2250 mg/60 kg of human. Calculated from daily food intake and body weight of the mice fed the MCD+HF diet, we mixed metformin at 0.1% in MCD+HF diet. The weights and food intakes in each group of rats were recorded every week. All animal procedures were performed in accordance with the standards set forth in the Guidelines for the Care and Use of Laboratory Animals at the Takara-machi campus of Kanazawa University

Effects of metformin on advanced stages of nonalcoholic steatohepatitis were evaluated as follows. The MCD+HF diet was fed to 8 week-old C57BL/6 mice for 8 weeks to create an advanced non-alcoholic steatohepatitis model. Then, mice were divided into two experimental groups that were fed for 4 weeks as follows: d) MCD+HF (MCD, fat 60%, *n* = 10), e) MCD+HF diet mixed with 0.1% metformin (MCD+Met, fat 60%, *n* = 10).

### Blood sampling and analysis

Blood samples were obtained from the tail vein at 16 and 20 weeks of age under anesthesia after a 4-h fast. Blood glucose was determined by the glucose-oxidase method using Freestyle (Kissei, Nagano, Japan). Blood samples were centrifuged and plasma was frozen at −80°C for subsequent measurement of the plasma triglyceride, total cholesterol (TC), and insulin levels. Serum TC and triglyceride concentrations were determined by an enzymatic method using the Cholesterol E-test and Triglyceride E-test (Wako Pure Chemical Industries, Osaka, Japan). Plasma insulin levels were determined with an ELISA kit (Mercodia, Uppsala, Sweden).

### Evaluation of Insulin Sensitivity

After 8 weeks, mice in all groups underwent an oral glucose tolerance test after a 4-hour fast. Two grams of glucose per kilogram of body weight was administered orally. Blood was drawn from a tail vein at 0, 30, 60, and 120 minutes for measurement of plasma glucose concentrations. An intraperitoneal insulin tolerance test was per- formed after a 4-hour fast by intraperitoneal administration of 0.5 unit insulin per kilogram of body weight. Blood was drawn from a tail vein at 0, 15, 30, 45, 60, and 120 minutes for measurement of plasma glucose concentrations.

### Measurement of liver triglyceride levels

Mice were sacrificed, and liver weight and the triglyceride content in liver tissue were measured. To quantify hepatic triglyceride content, the liver was lysed with buffer from a commercially available kit (TG E-test; Wako) and disrupted by sonication. The triglyceride content of the homogenate was then determined with the above kit, according to the manufacturer's instructions.

### Histological evaluation and immunohistochemistry

After 8, 10, and 12 weeks on each diet, animals were sacrificed and their livers were fixed in 10% buffered formalin and embedded in paraffin. The severity of hepatic histologic changes was assessed in hematoxylin and eosin- and Sirius red stained samples and blindly scored by a single pathologist who was unaware of the treatments for mice. Steatosis, inflammation, and fibrosis were semi-quantitatively evaluated according to the standard criteria of NASH grading and staging with minor modifications [18]. The degree of steatosis was scored as the percentage of hepatocytes containing lipid droplets. Inflammation was scored as: 0, no hepatocyte injury and inflammation, 1, mild focal injury, 2, noticeable injury, and 3, severe zone 3 hepatocyte injury and inflammation. Fibrosis was scored as: 0, no fibrosis, 1, pericellular and perivenular fibrosis, 2, focal bridging fibrosis, 3, extensive bridging fibrosis with lobular distortion, and 4, cirrhosis. Images of the histological slices of Sirius red stain were analyzed and captured under 100× magnification. Further Area of their fibrosis was morphometrically and statistically analyzed with Image J and SPSS software [19].

Slides were immunostained with monoclonal mouse anti-human α-smooth muscle actin (α-SMA) (Dako, Kyoto, Japan). This was followed by use of the immunoperoxidase technique using an Envision kit (Dako). Further their area of α-SMA was morphometrically and statistically analyzed with Image J and SPSS software [19].

### Hydroxyproline Assay

Hydroxyproline content of the liver was measured by a spectrophotometric assay as an assessment of liver collagen content. Liver tissue was homogenized in ice-cold distilled water (1 mL) using a polytron homogenizer. Subsequently, 125 µL of 50% trichloroacetic acid was added, and the homogenates were further incubated on ice for 30 minutes. Precipitated pellets were hydrolyzed for 24 hours at 110°C in 6N HCL.

After hydrolysis, the samples were filtered and neutralized with 10N NaOH, and the hydrolysates were oxidized with Chloramine-T (Sigma) for 25 minutes at room temperature. The reaction mixture was then incubated in Ehrich's perchloric acid solution at 65°C for 20 minutes and cooled to room temperature. Sample absorbance was measured at 560 nm. Purified hydroxyproline (Sigma) was used to set a standard. Hydroxyproline content was expressed as micrograms of hydroxyproline per gram liver [20].

### Real-time quantitative PCR

Total RNA was extracted from each liver using the RNeasy Mini kit (Qiagen, Tokyo, Japan), as described previously [21]. Real-time quantitative polymerase chain reaction (PCR) was performed for transforming growth factor-β (*Tgfb*), α1(I) procollagen a2 (*Col1a2*), plasminogen activator inhibitor-1 (PAI-1: encoded by *Serpine-1* gene), sterol regulatory element-binding protein-1c (*Srebp1c*), fatty acid synthase (*Fas*), cytochrome P450 2e1 (*Cyp2e1*), apolipoprotein B (*Apob*), microsomal triglyceride transfer protein (*Mttp*) and hemeoxygenase (*Hmox1*) mRNA using the ABI Prism 7900 Sequence Detection System (Applied Biosystems, Foster City, CA, USA). The primer sets and TaqMan probes for *Col1a2*, *Serpine1*, *Fas*, *Cyp2e1*, *Apob*, *Mttp* and *Hmox1* are proprietary to Applied Biosystems (Assay-on-Demand gene expression product). The primer sets and TaqMan probes for *Tgfb* and *Srebp1c* were designed with Primer Express (ver. 1.5; Applied Biosystems). The forward primers were 5′-TTCCTGGCGTTACCTTGGT-3′ for *Tgfb* and 5′-GGGCAGCTCTGTACTCCTTCAA-3′ for *Srebp1c*. The reverse primers were 5′-GCCACTGCCGGACAACT-3′ for *Tgfb* and 5′-GCTAAGCTGTCCCGCAGGTA-3′ for *Srebp1c*. The TaqMan probes were 5′-TACGCCTGAGTGGCTGTCTTTTGA-3′ for *Tgfb* and 5′-AGCCAGCCTGGCCATCTGTGAGA-3′ for *Srebp1c*. The amount of DNA available for PCR in the different samples and target gene sequence expression were normalized with respect to the expression of an endogenous control, 18S ribosomal RNA (18S rRNA TaqMan Control Reagent kit; Applied Biosystems)(*Col1a2*, *Serpine1*, *Fas*, *Cyp2e1*, *Tgfb* and *Srebp1c*) and beta-actin (TaqMan Control Reagent kit; Applied Biosystems) (*Apob*, *Mttp*). The PCR conditions were one cycle at 50°C for 2 min and 95°C for 10 min, followed by 50 cycles at 95°C for 15 s and 58°C for 1 min.

### Western blotting

Western blotting was performed with iBlot Western Detection Kit (life technologies) as we previously reported [22]. The antibodies for PAI-1, FAS and APOB were purchased from Abcam.

### DNA microarray analysis

A DNA microarray analysis was performed with an Affymetrix GeneChip (Mouse Genome 430 2.0 Array; Affymetrix, Santa Clara, CA, USA). Briefly, 2 µg of total RNA was used as a template for cDNA synthesis with the GeneChip Expression 3′-Amplification One-Cycle cDNA Synthesis kit (Affymetrix), and biotin-labeled cRNA was synthesized with GeneChip Expression 3′-Amplification Reagents (Affymetrix). After generating the hybridization cocktails, hybridization to the DNA microarray and fluorescent labelling were performed with a GeneChip Hybridization Wash and Stain kit (Affymetrix). The microarrays were subsequently scanned with the GeneChip Scanner 3000 MegAllele System (Affymetrix). Our DNA chip data was accepted as approval number GSE35961 in GEO (Gene Expression Omnibus).

### Microarray data analysis

Data analysis was conducted with the GeneChip Operating System 1.1 (Affymetrix). Detection, signal, signal log ratio, and change were obtained from the GeneChip Operating System (GCOS) with default settings after global normalization was performed to make the average intensity of all probe sets equal to 100. The fold-change value was calculated from the signal log ratio. Probe sets with expression changes labelled as change call “I” or “D” by GCOS were referred to as “regulated probe sets.” “Regulated probe sets” with more than a 1.5-fold difference in expression were extracted to choose differentially expressed probe sets among the 45,101 probe sets represented on the array. Among them, the probe sets showing significant expression changes (p<0.05; Student's *t*-test) in the analysis of individual samples (*n* = 4) were chosen for further analysis.

Principal component analysis (PCA) or hierarchal clustering was performed with Spotfire DecisionSite 9.1 (Spotfire, Somerville, MA, USA).

### Canonical pathway analysis/gene-to-gene network analysis

Canonical pathway and gene-to-gene network analyses were performed using Ingenuity Pathways Analysis (ver. 5.5.1; Ingenuity Systems, www.ingenuity.com, Redwood City, CA, USA). The extracted data set containing differentially expressed probe sets and corresponding values of the signal log ratio was uploaded into the application. Each probe set was mapped to its corresponding gene object in the Ingenuity Pathways Knowledge Base.

A canonical pathway analysis identified the pathways from the Ingenuity Pathways Analysis library of canonical pathways that were most significant to the differentially expressed genes. Genes that were associated with a canonical pathway in the Ingenuity Pathways Knowledge Base were considered for analysis. The significance of the association between the genes and the canonical pathway was measured in two ways: 1) the ratio of the number of genes from the data set that map to the pathway divided by the total number of genes that map to the canonical pathway is displayed, and 2) Fischer's exact test was used to calculate a p-value determining the probability that the association between the genes in the dataset and the canonical pathway was explained by chance alone. Canonical pathways associated with the differentially expressed genes were extracted with the calculated p-value cut-off of 0.05.

### Statistical analysis

All results are expressed as the mean ± standard error of the mean. Data were analyzed using a one-factor analysis of variance to compare the means of all groups. Between-two group differences in continuous variables were assessed by a univariate analysis with Student's *t*-test. One-way ANOVA was used for the comparison of more than two groups, followed by Tukey-Kramer post hoc test. P-value<0.05 was considered to indicate statistical significance. All calculations were performed with the Stat View software (ver. 5.0; SAS Institute Inc., Cary, NC, USA).

## Results

### Metabolic parameters

**Figure 1:**
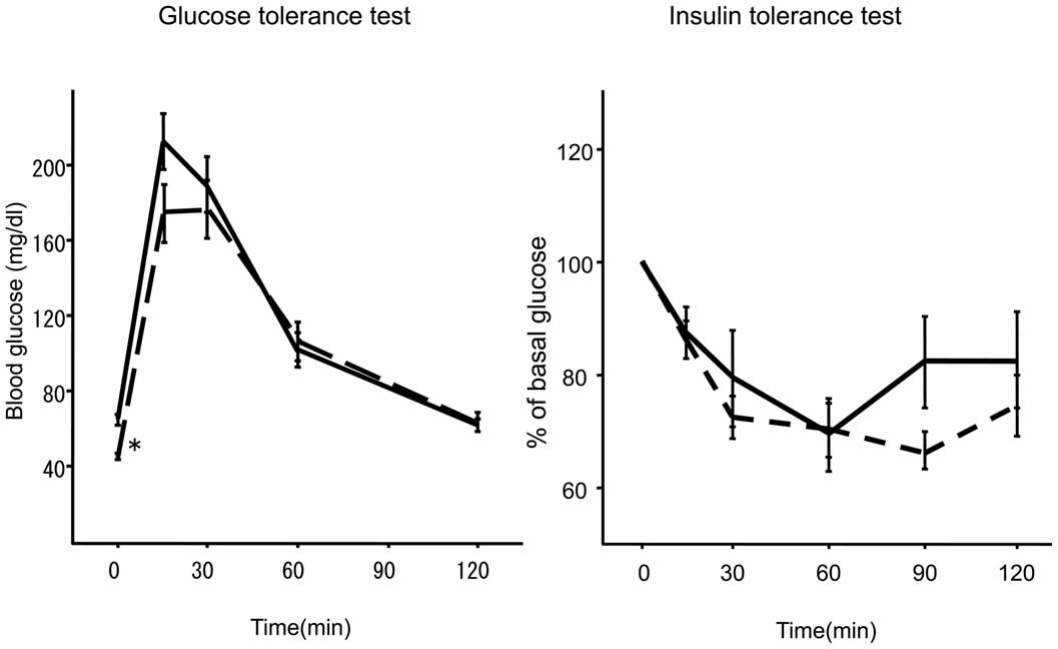
Intraperitoneal glucose tolerance test (A) and insulin tolerance test (B). Black line, methionine- and choline-deficient+high fat diet (MCD+HF, *n* = 15). Short dashed line, MCD+HF diet mixed with 0.1% metformin (MCD+Met; *n* = 20). *p<0.05, vs. MCD+HF diet group.

Body and liver weights and lipid levels after 8 weeks in each group are shown in Table 1. Co-administration of metformin did not affect body weight, physical appearance, or behavior of the mice. Amount of food consumption was also unchanged between MCD+HF groups and MCD+HF+Met groups. Metformin significantly decreased the liver weights in mice that received the MCD+HF diet (p<0.05, vs. mice fed MCD+HF diet) without affecting body weight. Co-administration of metformin increased serum TC levels. Metformin decreased fasting blood glucose levels without affecting serum insulin levels (Table 1). We conducted intraperitoneal glucose and insulin tolerance tests at 8 weeks to evaluate the effect of metformin on glucose tolerance and insulin sensitivity, respectively (Fig. 1). Although basal glucose levels were significantly lower in mice treated with metformin, the glucose increase after a glucose challenge and the glucose decrease after an insulin challenge did not differ between the groups. These findings suggest that metformin exerts a minimum effect on glucose tolerance and insulin sensitivity in this non-diabetic mouse model of steatohepatitis.

**Table 1:** Metabolic parameters in the methionine- and choline- deficient (MCD) diet-induced mice of nonalcoholic steatohepatitis after 8 weeks of metformin (Met) treatment.

Parameters	MCD+HF	MCD+HF+Met
Body weight (g)	15.2±0.2	14.6±0.3
Liver weight (mg)	811±31	638±33*
Food consumption (mg/day)	214±15	204±43
Fasting blood glucose (mg/dL)	63.7±8.9	51.1±4.7*
Fasting serum insulin (pg/mL)	495±17	550±44
Fasting serum triglycerides (mg/dL)	47.9±3.2	49.8±2.6
Serum total cholesterol (mg/dL)	20.8±0.8	47.4±1.9*

Data are means ± standard error of the mean.

* p<0.05 versus untreated Met diet fed mice.

### Metformin ameliorates liver pathology in a NASH dietary mouse model

**Figure 2:**
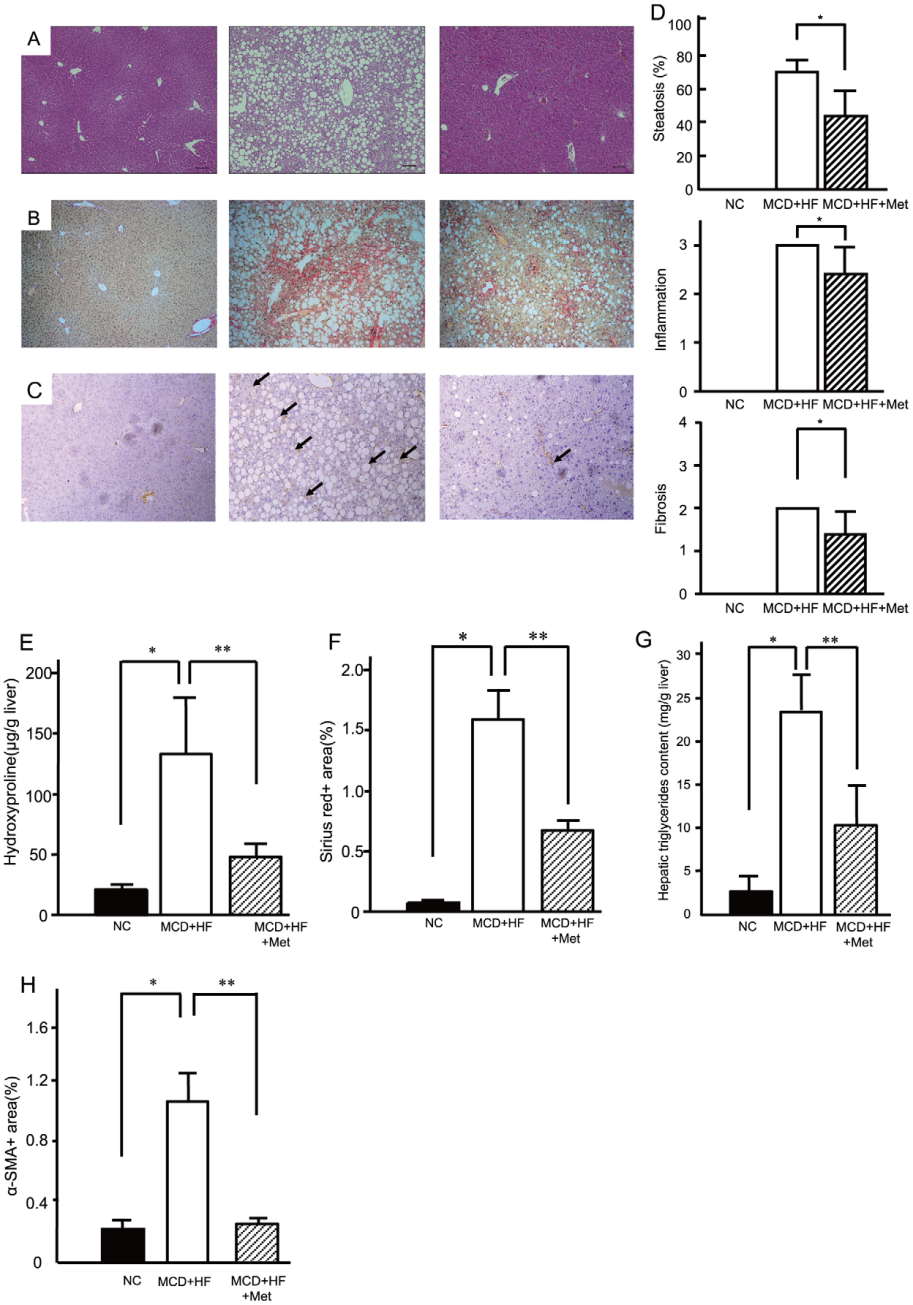
Metformin ameliorated the pathology in a non-alcoholic steatohepatitis dietary model. Representative photomicrographs show the effects of normal chow (NC, *n* = 10), the methionine- and choline-deficient+high fat diet (MCD+HF, *n* = 15), and the MCD+HF diet mixed with 0.1% metformin (MCD+HF+Met; *n* = 20) on the liver histology in C57BL/6 mice. Mice were fed the diets for 8 weeks. Paraffin-embedded sections were stained with (A) hematoxylin and eosin or (B) sirius red and (C) immunohistochemically stained with anti-α-smooth muscle actin. Bar, 20 µm. Original magnification, ×100. (D) Blinded observers scored the hematoxylin-and-eosin-stained sections for steatosis and inflammation severity; azan-stained samples were scored for fibrosis. The scoring criteria are described in the Materials and Methods. Values are means ± standard error of the mean. *p<0.05, vs. MCD+HF diet group. (E) Hepatic hydroxyproline (F) morphometric analysis of liver fibrosis of sirius red stain (G) Metformin improved hepatic triglyceride content in diet-induced non-alcoholic steatohepatitis model mice. (H) Area of α-SMA. Black bar, normal chow (NC, n = 10). White bar, the methionine- and choline-deficient+high fat diet (MCD+HF, *n* = 15). Mosaic Bar, the MCD diet mixed with 0.1% metformin (MCD+HF+Met; *n* = 20). Values are the mean ± standard error. *p<0.05, vs. normal chow. **p<0.05, vs. the MCD+HF diet group.

The histological findings at 8 weeks of treatment are shown in Fig. 2A and B. The MCD diet caused marked macrovesicular steatosis with focal lymphocytic infiltration, hepatocellular drop-outs, intense lobular inflammation, and prominent perivenular and pericellular fibrosis in zone 3 of the 8-week-old mice livers. The histological score based on the current diagnostic criteria at 8 weeks of treatment is summarized in Fig. 2D. Co-administration of metformin significantly ameliorated the MCD diet-induced steatosis, inflammation, and fibrosis. Moreover, metformin inhibited the elevation of hepatic hydroxyproline contents induced by MCD+HF diet (Fig. 2E). In addition, metformin decreased the positive area of Sirius Red stain, a representative staining for the connective tissues (Fig. 2F). Indeed, metformin treatment significantly improved MCD diet-induced hepatic triglyceride accumulation in C57BL/6 mice (Fig. 2G).

### Metformin decreases activated stellate cells and downregulates mRNA expression of fibrogenic genes in a NASH dietary mouse model

We performed an immunohistochemical analysis of α-SMA after 8 weeks to investigate the activation of hepatic stellate cells, which play central roles in liver fibrosis. Representative photomicrographs of liver sections stained with anti-α-SMA antibody are shown in Fig. 2C. Activated stellate cells, which express α-SMA and are therefore also called myofibroblast-like cells, showed prominent proliferation in the liver of mice fed the MCD diet. Metformin treatment reduced α-SMA-positive cells in the livers of mice that received the MCD diet (Fig. 2H).

### Global gene expression profile in the livers of individual mice

**Figure 3:**
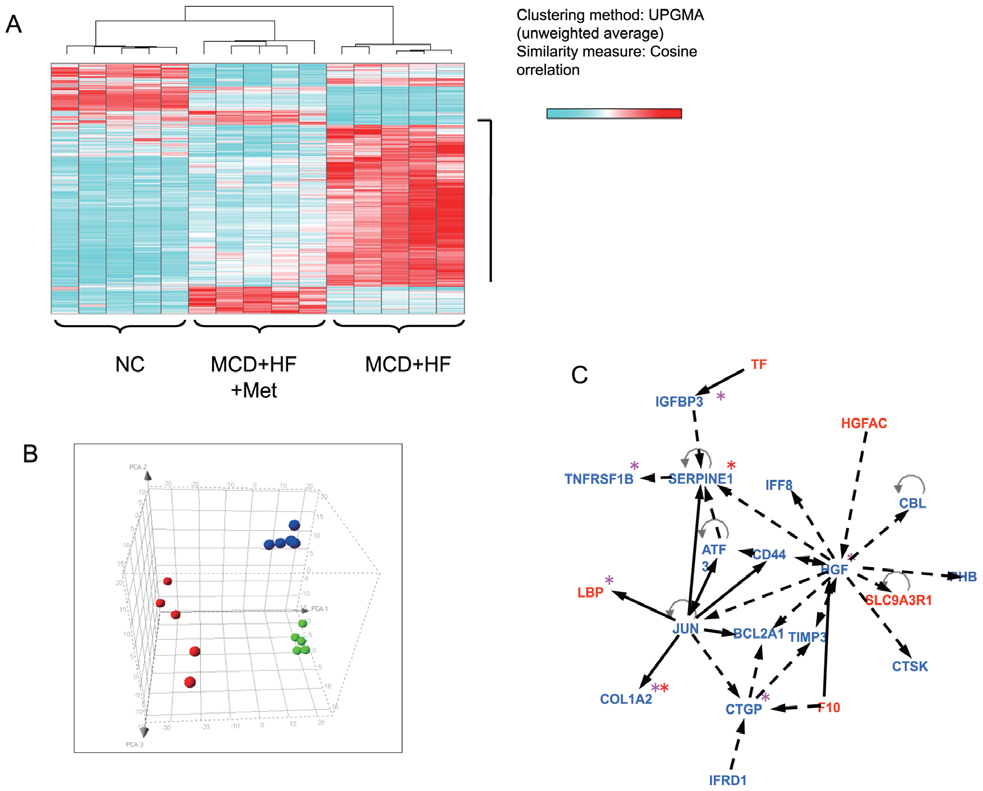
Comprehensive gene expression analyses in livers of mice treated with metformin. (A) Gene expression profile analysis using materials from individual animals and performed unsupervised hierarchical clustering of all sets of expression data with the 792 genes. The results clearly showed that mice that had been treated with metformin were clustered together with normal chow and could be separated from no treatment. (B) Principal component analysis using the same 792 genes dataset showed a remarkable shift in the distribution of mice treated with metformin compared with no treatment. Green, normal chow group; Red, the methionine- and chorine-deficient (MCD) diet+high fatgroup; Blue, the MCD+HF diet mixed with 0.1% metformin group. (C) Gene-to-gene network analysis was used to investigate molecular relationships between differentially expressed genes included in Hepatic Fibrosis/Hepatic Stellate Cell Activation pathway. Red asterisk (*): NASH related genes. Pink asterisk (*): hepatic fibrosis related genes. Red: genes up-reguleted by metformin treatment. Blue: genes down-regulated by metformin treatment.

This analysis identified 792 genes that showed at least a 1.5-fold difference in expression following metformin treatment. We performed a gene expression profile analysis using materials from 15 individual animals and performed unsupervised hierarchical clustering of all 15 sets of expression data with the 792 genes to examine the relevance of these subtle gene expression changes. The results showed that mice treated with metformin were clustered together with those who were fed normal chow and could be separated from the untreated (Fig. 3A). Furthermore, PCA using the same 792 genes data set showed a remarkable shift in the distribution of mice treated with metformin compared with untreated mice (Fig. 3B). Moreover, a canonical pathway analysis of the expression profile revealed that metformin effected significant alterations in gene expression across at least 11 metabolic pathways, including those involved in fatty acid and amino acid metabolism (Table 2). A gene network based on molecular relationships between differentially expressed genes included in the hepatic fibrosis/hepatic stellate cell activation pathway was built from the biological relationships stored in the Ingenuity Pathways Knowledge Base (Fig. 3C). Metformin treatment ameliorated activity in the hepatic fibrosis/hepatic stellate cell activation pathway, such as *Serpine-1*, *collagen Ia 2* (*Col1a2*), endothelin receptor type B (*Ednrb*), hepatic growth factor (*Hgf*), connective tissue growth factor (*Ctgf*), tissue inhibitors of matrix metalloproteinase (*Timp*), tumor necrosis factor receptor superfamily 1B (*Tnfrsf1b*), and insulin like growth factor binding protein 3 (*Igfbp3*). Metformin prevented expression of inflammation and fibrosis genes cooperatively as well as those of fatty acid metabolism in the liver of the NASH dietary mouse model.

**Table 2:** Canonical pathways analysis identified the pathways from the Ingenuity Pathways Analysis library of canonical pathways were most significant to the differentially expressed genes.

Pathway	1.5 fold up-regulated gene (MCD+HF+Met vs MCD+HF)	1.5 fold down-regulated gene (MCD+HF+Met vs MCD+HF)	−log_10_(p value)
Complement and Coagulation Cascades	MBL2, C9	C3AR1	6.21
Leukocyte Extravasation Signaling	-	MMP12, ARHGAP9, ITGB2, CYBB, CYBA, CD44, RAC2, TIMP3, MSN, TIMP2	4.07
Acute Phase Response Signaling	SERPING1, MBL2, C4B, LBP C9	JUN, HMOX1, PAI1, TNFRSF1B, TF	2.76
Hepatic Fibrosis/Hepatic Stellate Cell Activation	LBP	COL1A2, EDNRB, HGF, CTGF, TIMP2, TNFRSF1B, IGFBP3	2.76
Linoleic Acid Metabolism	CYP2B10, CYP2B13, CYP2C38	CYP4A12, CYP1B1	2.47
LPS/IL-1 Mediated Inhibition of RXR Function	ABCC4, FMO2, FABP4, TNFRSF1B	GSTT1, FABP7, LBP, JUN, ACOX1	2.37
Antigen Presentation Pathway	-	HLA-DQB2, CD74, HLA-DQA1	2.07
Tryptophan Metabolism	CCBL1, CYP2C44, CYP2C70, CYP2C50, HAAO	CYP1B1	1.76
Arachidonic Acid Pathway	CYP2C44, CYP2C70, PLA2G12B, CYP2C50	PLA2G4A, CYP1B1	1.68
PPARa/RXRa Activation	CYP2C44, CYP2C70, CYP2C50	ADCY7, JUN, LPL, ACOX1	1.60
Metabolism of Xenobiotics by Cytochrome P450	CYP2C44, CYP2C70, CYP2C50, GSTT1	CYP1B1	1.54

### Metformin downregulates lipogenic and fibrogenic gene expression in the NASH dietary mouse model

**Figure 4:**
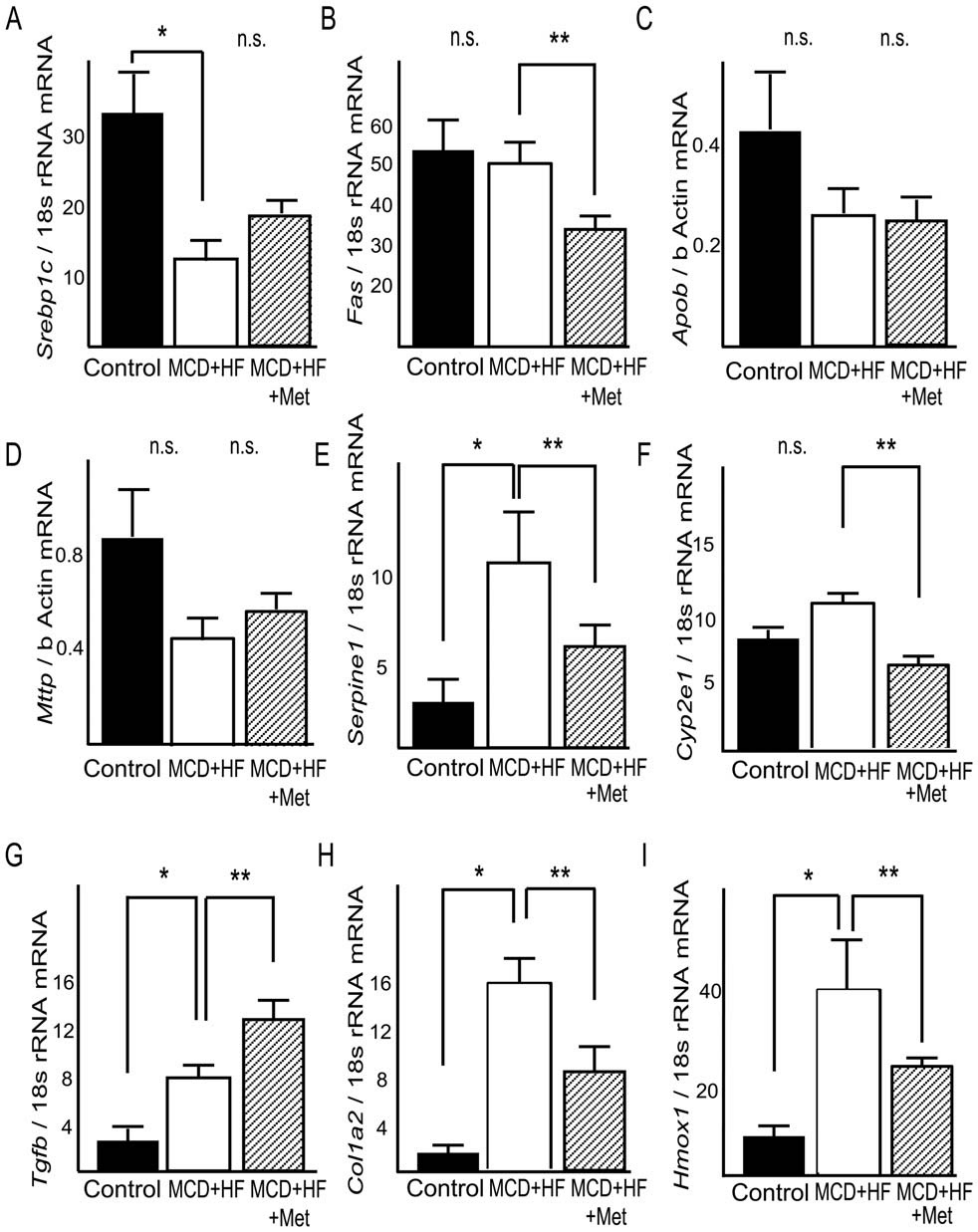
Effects of metformin on expression of genes involved in steatosis, inflammation, and fibrosis in the liver of mice fed a MCD+HF diet. Real-time quantitative polymerase chain reaction was used to measure the hepatic expression of genes encoding (A) sterol regulatory element-binding protein-1c (*Srebp1c*), (B) fatty acid synthase (*Fas*), (C) apolipoprotein B (*Apob*), (D) microsomal triglyceride transfer protein (*Mttp*), (E) plasminogen activator 1 (*Serpine1*), (F) cytochrome P450 2e1 (*Cyp2e1*), (G) transforming growth factor-β (*Tgfb*), (H) procollagen1a2 (*Col1a2*), (I) hemeoxigenase1 (*Hmox1*). Results were normalized against 18S rRNA (*Srebp1c*, *Fas*, *Serpine1*, *Cyp2e1*, *Tgfb*, *Col1a2*, *Hmox1*) and beta-actin (*Apob,Mttp*). Values are means ± standard error. *p<0.05, vs. normal chow. **p<0.05, vs. MCD+HF diet group.

Because the gene expression profile results indicated that metformin prevents the progression of NASH by altering genes expressed during fibrosis and fatty acid metabolism, we assessed the effect of metformin on mRNA expression of these genes by real-time PCR. Metformin inhibited *Fas* hepatic mRNA expression to 60% in the livers of the NASH dietary mouse model (p<0.05, vs. MCD diet; Fig. 4B). Metformin also inhibited hepatic mRNA expression of *Serpine1* to 42%, *Cyp2e1* to 55%, and *Col1a2* to 56% in the livers of MCD-induced steatohepatitis model mice (p<0.05, vs. MCD diet; Fig. 4F, 4G and 4H). Moreover, metformin also coordinately ameliorated downregulated genes for oxidative stress-associated proteins, such as *Hmox1*, in the livers of MCD-induced steatohepatitis model mice (p<0.05, vs. MCD diet; Fig. 4I).

### Metformin downregulates protein levels of PAI-1 in the NASH dietary mouse model

**Figure 5:**
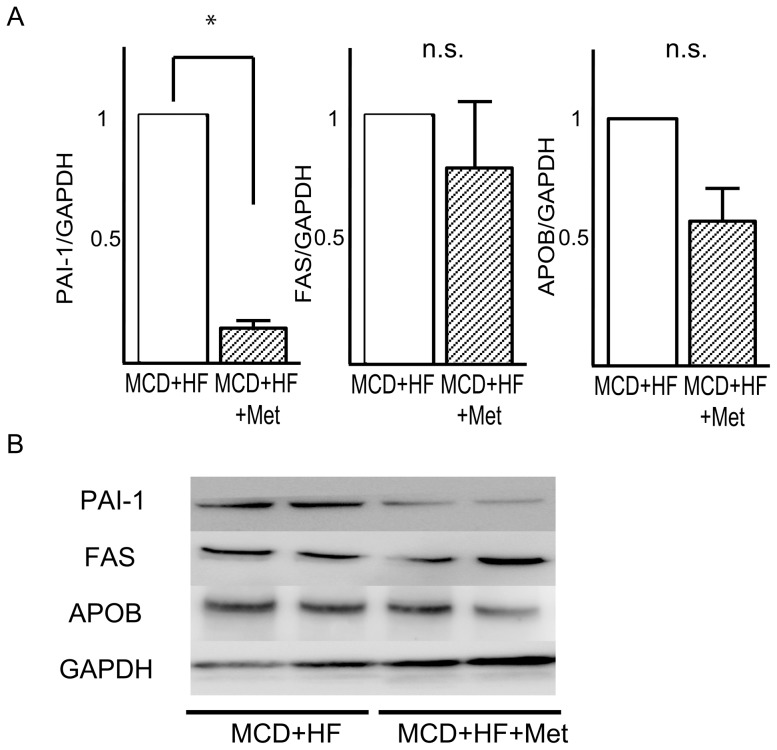
Effects of metformin on the levels of proteins involved in lipid metabolism in the liver of mice fed a MCD+HD diet. (A) Quantitative data from densitometric analysis of Western blots from three samples. (B) Representative blots for PAI-1, FAS, and, APOB are shown. GAPDH is used as a control for protein loading. Values are the mean ± standard error. *p<0.05 versus the MCD+HF diet group.

Next, we examined protein levels of PAI-1, FAS, and APOB by using Western blotting, Consistent with the result of realtime PCR, protein levels of PAI-1 were significantly decreased by metformin (Fig. 5A and 5B). However, FAS protein levels were not significantly altered by metformin treatment.

### Metformin reverses steatosis and inflammation of the dietary mouse model in the advanced stages of NASH

To determine whether metformin improves pre-existing NASH in mice, we examined the therapeutic effect of metformin on advanced stage NASH in the model mice. The results of body and liver weights and lipid levels after 4 weeks of treatment in each group are shown in Table 3. Co-administration of metformin inhibited weight gain and improved glycemic levels compared with metformin-untreated mice. Serum insulin levels were similar in each group. Food consumption was unchanged by metformin.

**Table 3:** Metabolic parameters in nonalcoholic steatohepatitis model mice at 4 weeks of metformin (Met) treatment after feeding 8-week-methionine- and choline-deficient (MCD) diet.

Parameters	MCD+HF	MCD+HF+Met
Body weight (g)	14.6±0.8	12.5±0.7*
Liver weight (mg)	991±58	838±68*
Food consumption (mg/day)	206±27	217±13
Fasting blood glucose (mg/dL)	61.0±7.0	33.2±4.0*
Fasting serum insulin (pg/mL)	467±37	490±31
Fasting serum triglycerides (mg/dL)	47.6±2.9	49.3±3.9
Serum total cholesterol (mg/dL)	33.7±5.2	49.9±5.7*

Data are means ± standard error of the mean.

* p<0.05 versus untreated Met diet fed mice.

**Figure 6:**
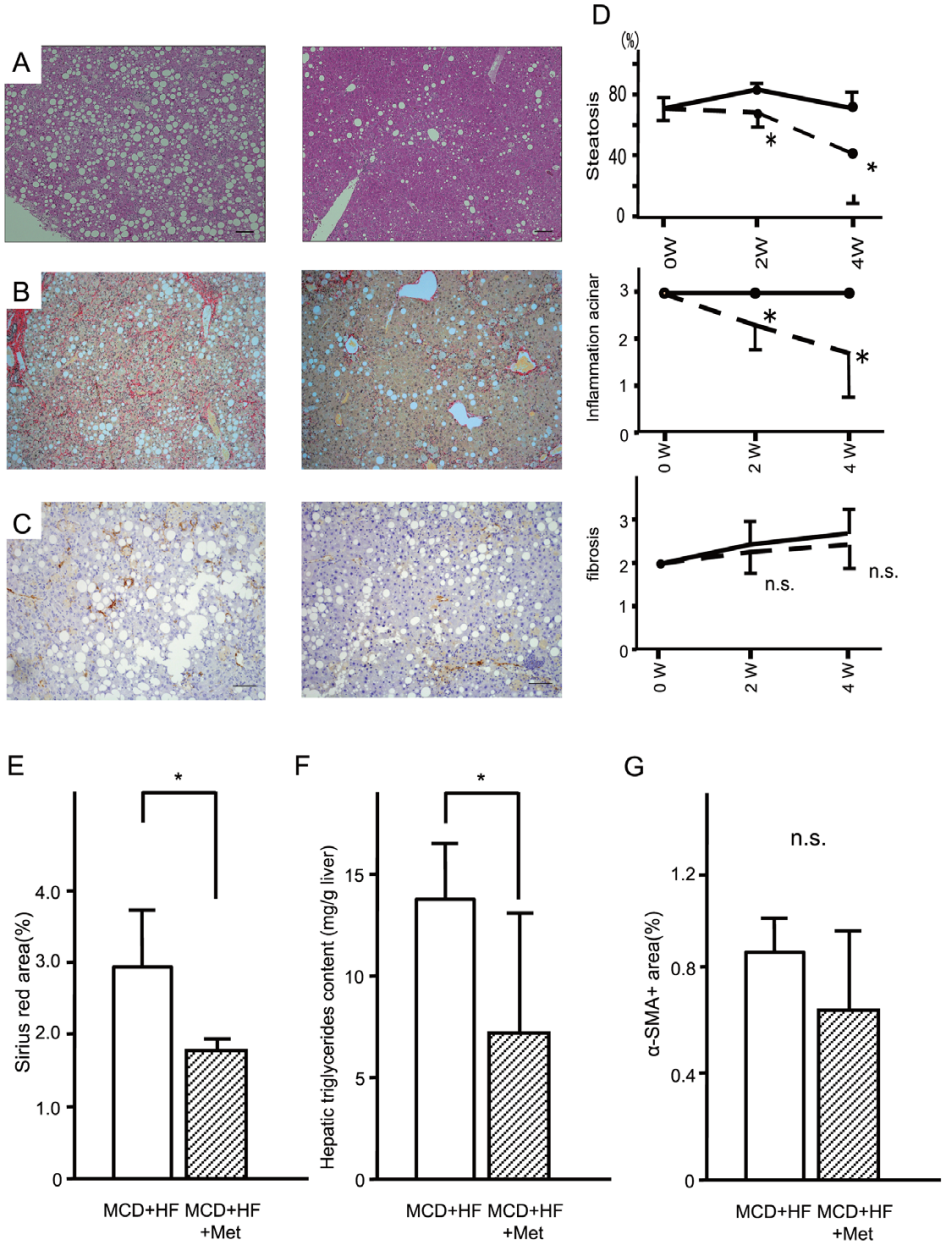
Metformin reversed steatosis and inflammation of the advanced stages of nonalcoholic steatohepatitis in mice. Representative photomicrographs show the effects of the methionine- and choline deficient plus high fat diet (MCD+HF, n = 10) and the MCD+HF diet mixed 0.1% metformin (MCD+HF+Met; n = 10). Mice fed the diets for 4 weeks from the advanced stages of steatohepatitis. Paraffin-embedded sections were stained with (A) hematoxylin–eosin, (B) Sirius Red and (C) immunohistochemically stained with anti-α-smooth muscle actin. Bar, 20 µm. Original magnification, ×100. (D) Metformin improved hepatic triglyceride content of diet-induced non-alcoholic steatohepatitis. Mice were fed the methionine- and choline deficient+high fat diet (MCD+HF, n = 10) and the MCD+HF diet mixed 0.1% metformin (MCD+HF+Met; n = 10). Values are the mean ± standard error of the mean. *p<0.05 versus the MCD+HF diet. (E) Morphometric analysis of liver fibrosis of sirius red stain(%). (F) Area of alpha-SMA(%). (G) Metformin improved hepatic triglyceride content of diet-induced non-alcoholic steatohepatitis. White Bar, continuous methionine- and choline deficient+high fat diet (MCD+HF, n = 5). Mosaic Bar, the MCD+HF diet mixed 0.1% metformin (MCD+HF+Met; n = 10). Values are the mean ± standard error. *p<0.05 versus the MCD+HF diet group.

As shown in Fig. 6, metformin ameliorated macrovesicular steatosis with focal lymphocytic infiltration and hepatocellular drop-outs, and intense lobular inflammation at 4 weeks (Fig. 6D). Liver fibrosis score (Fig. 6D) and area of α-SMA (Fig. 6F) were unaffected. However, metformin significantly decreased the positive area of Sirius Red staining (Fig. 6E). Metformin also significantly improved MCD diet-induced hepatic triglyceride accumulation in C57BL/6 mice (p<0.05, Fig. 6G).

**Figure 7:**
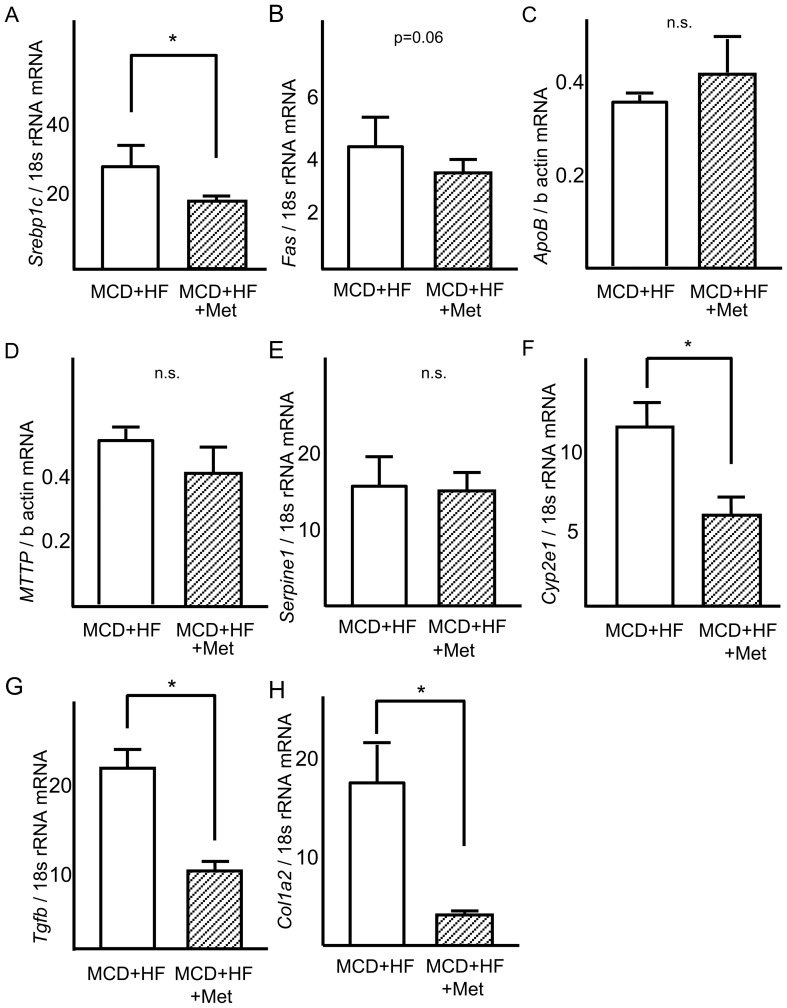
Reverse effects of metformin on expression of genes involved in steatosis, inflammation, and fibrosis in the liver of mice with the advanced stages of nonalcoholic steatohepatitis. Real-time quantitative polymerase chain reaction was used to measure the hepatic expression of genes encoding (A) sterol regulatory element-binding protein-1c (*Srebp1c*), (B) fatty acid synthase (*Fas*), (C) apolipoprotein B (*Apob*), (D) microsomal triglyceride transfer protein (*Mttp*), (E) plasminogen activator 1 (*Serpine1*), (F) cytochrome P450 2e1 (*Cyp2e1*), (G) transforming growth factor-β (*Tgfb*), (H) procollagen1a2 (*Col1a2*). Results were normalized against 18S rRNA (*Srebp1c*, *Fas*, *Serpine1*, *Cyp2e1*, *Tgfb*, *Col1a2*) and beta-actin(*Apob,Mttp*). Values are means ± standard error. *p<0.05 versus the MCD+HF diet group.

**Figure 8:**
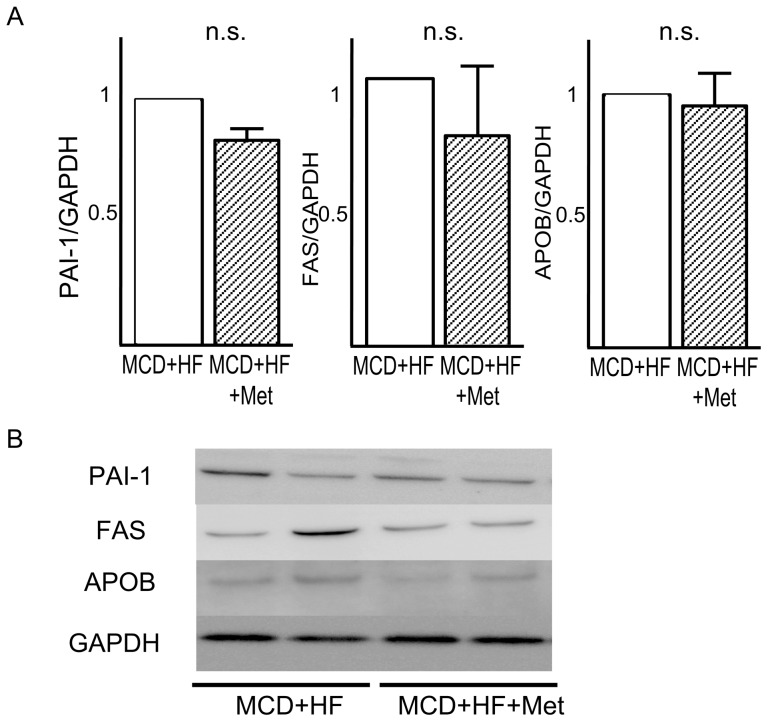
Effects of metformin on the levels of proteins involved in lipid metabolism in the liver of mice with the advanced stages of nonalcoholic steatohepatitis. (A) Quantitative data from densitometric analysis of Western blots from three samples. (B) Representative blots for PAI-1, FAS, and, APOB are shown. GAPDH is used as a control for protein loading. Values are the mean ± standard error. *p<0.05 versus the MCD+HF diet group.

Metformin inhibited hepatic mRNA expression of *Srebp1c* to 67% and that of *Cyp2e1* to 45% in the livers of mice fed the MCD diet (*P*<0.05, vs. MCD diet for both variables; Fig. 7A and 7F). Moreover, metformin inhibited hepatic mRNA expression of *Tgfb* to 33% and *Col1a2* to 17% in the livers of mice fed the MCD diet (P<0.05, vs. MCD diet for both variables; Fig. 7G and 7H). Protein levels for PAI-1, FAS and APOB were unchanged by metformin treatment (Fig. 8).

## Discussion

Previous studies evaluating the effects of metformin on NAFLD liver pathology are limited. In genetically obese model ob/ob mice, Lin *et al.* reported that metformin was effective at reversing fatty liver, probably via reduced hepatic expression of tumor necrosis factor, which promotes hepatic lipid accumulation and ATP depletion [15]. In humans, Marchesini *et al.* showed that long-term metformin treatment significantly reduced mean transaminase concentrations and decreased liver volume by 20% [16]. Although metformin prevents fat accumulation in a simple fatty liver, whether metformin ameliorates hepatic inflammation and fibrosis in steatohepatitis remains unclear. We demonstrated that metformin prevents and reverses pathological development in a NASH dietary mouse model. This is the first experimental evidence that metformin can prevent and reverse the development of not only steatosis but also inflammation in the liver of a NASH model.

High levels of insulin cause fatty liver in insulin resistant states, suggesting that mice with type 2 diabetes manifest selective hepatic insulin resistance: insulin fails to suppress gluconeogenesis but continues to activate lipogenesis [23]. Whether metformin improves insulin resistance remains controversial [24], [25]. Basu *et al.* reported that metformin at a dose of 2000 mg/day for 4 months did not improve insulin-induced stimulation of glucose disappearance and did not improve impairment of insulin-induced suppression of hepatic glucose production [25]. Indeed, in the present study, metformin seemed to exert a minimal effect on glucose tolerance and insulin sensitivity, as shown by the intraperitoneal glucose and insulin tolerance test results. Metformin significantly lowered fasting glucose levels without altering fasting insulin levels, suggesting that metformin directly suppressed hepatic gluconeogenesis independently of an insulin signaling pathway, possibly by activating AMPK [7], [26].

We conducted a global gene expression profile analysis to search for direct actions of metformin in addition to the improved insulin resistance. NASH model mice that had been treated with metformin were clustered together with control mice and could be separated from NASH model mice that did not receive metformin treatment. Indeed, a PCA using the gene dataset showed a remarkable shift in the distribution of NASH model mice treated with metformin compared with untreated mice. A canonical pathway analysis of the expression profiles revealed that metformin effected significant alterations in gene expression across at least 11 metabolic pathways, including those involved in fatty acid and amino acid metabolism, such as the hepatic fibrosis/hepatic stellate cell activation pathway.

Metformin decreased gene expression levels for *Fas*. FAS is a key enzyme in fatty acid biosynthesis and is believed to be a determinant of the maximal capacity of the liver to synthesize fatty acids by *de novo* lipogenesis, because FAS catalyzes the last step in the fatty acid biosynthetic pathway [27]. The metformin-induced reduction of hepatic *Fas* mRNA levels may have contributed to the improved hepatic steatosis in our model, as reported previously [3].

The hepatic export of triglyceride and cholesterol is a major determinant of steatosis as well as de novo lipogenesis. In the present study, neither treatment with metformin nor MCD+HF diet altered mRNA and protein levels for ApoB48, suggesting that hepatic lipid export does not seem to be a major determinant of hepatic steatosis altered by metformin treatment or MCD+HF diet. However, further direct studies are needed to assess whether metformin alters lipid export from the liver.

Metformin treatment decreased levels of gene expression for *Serpine-1* in the livers of MCD+HF diet-induced steatohepatitis in mice. This result is compatible with previous studies in hepatoma cell lines [28] and in the plasma of patients with type 2 diabetes [29]. PAI-1 is an acute phase protein expressed under conditions of inflammation [30]–[32] and contributes to the development of organ fibrosis by inhibiting matrix metalloproteinase activity [33]. Furthermore, recent work has indicated that *Serpine1* may have lipid metabolism activity. Ma *et al.* found that *Serpine1* ^−/−^ mice were protected from hepatic lipid accumulation in a model of high-fat/high-carbohydrate diet-induced obesity [34]. Indeed, the PAI-1/plasmin system also acts at the liver and might be functionally important in liver extracellular matrix remodeling in the NASH experimental model [3]. Bergheim *et al.* showed that metformin ameliorated liver pathology by suppressing *Serpine1* expression in an experimental alcoholic hepatitis mice model [35]. Importantly, they reported that metformin-mediated suppression of PAI-1 is independent of AMPK pathway activation. In the current study, microarray analysis showed no significant alteration of gene expression profiles in the pathways involved in AMPK/SREBP1c nor beta-oxidation (data not shown). These data suggest that metformin improved liver pathology, at least partly, by suppressing production of PAI-1 through the AMPK-independent pathway. Further studies are needed to clarify the AMPK-independent pathways that mediate the suppressive effect of metformin on PAI-1 production in the hepatocytes.

Metformin also coordinately altered genes associated with acute phase response signaling such as hemeoxygenase (*Hmox1*) in the livers of MCD-induced steatohepatitis model mice. Heme oxygenase 1 is a reductase enzyme that exerts anti-inflammatory and anti-fibrotic effects [36]. These findings suggest that metformin may improve steatohepatitis pathology by its anti-inflammatory profile.

One limitation of this study is that it is not fully determined whether metformin reverses hepatic fibrosis in diet-induced model of NASH. We performed the measurement of hydroxyproline contents and staining of Sirius Red to quantitate hepatic fibrosis objectively. In the preventive experiments, these results clearly indicated that metformin significantly prevented hepatic fibrosis induced by MCD+HF diet. On the other hand, in the reversal experiments, we could not observe longer time outcome of the liver histology because administration of MCD+HF diet for more than 8 weeks causes malnutrition-related death in mice. However, decrease in the positive area of Sirius Red and down-regulation of down-regulation of the genes involved in fibrogenesis such as *Tgfb* and *Col1a2* in the liver (Fig. 7G and 7H) suggest that metformin has the potential to reverse the pre-existing fibrosis. Additional experiments using other animal models for NASH are necessary to confirm whether metformin reverses the pre-exist fibrosis.

Generally, drug administration experiments may lose body weight in mice due to their toxic effects. However, in the current study, food consumption was unchanged by treatment with metformin in both preventive and reversal experiments, indicating that the present doses of metformin were not toxic in terms of food intake. These data suggest that metformin exerted beneficial effects on MCD+HF diet-induced steatohepatitis independently of food intake.

We showed that treatment with metformin rather increased the levels of serum cholesterol (Table 1 and 3). As we previously reported [3], steatohepatitis induced by MCD+HF diet results in lower serum cholesterol levels probably due to impaired hapatic function of cholesterol synthesis. Thus, in the present study, we interpreted that metformin ameliorated liver function together with liver pathology, and thereby recovered the decrease in serum cholesterol levels.

Treatment with metformin for 4 weeks significantly decreased mRNA levels for *Srebp1c* and *Tgfb* in the reverse experiment, whereas this decrease was not observed in the preventive experiment. The data of triglyceride content and hydroxyproline indicated that both hepatic steatosis and fibrosis were dramatically improved by treatment with metformin for 8 weeks in the preventive experiments. Hence, altered hepatic expression of *Srebp1c* and *Tgfb* may be caused by attenuation of steatosis and fibrosis. That is, normalized liver pathology masked the direct effects of metformin on *Srebp1c* and *Tgfb* through the secondary changes in the preventive experiments. Other possibility of altered gene expression may be existence of the alternative pathway leading to steatosis and fibrosis of the liver. Bergheim et al. showed that metformin ameliorated liver pathology by suppressing PAI-1expression in an experimental alcoholic hepatitis mice model [35]. In this paper, genetic deletion of PAI-1 ameliorated not only fibrosis, but also steatosis and inflammation in the liver. In the present study, treatment with metformin for 4 weeks significantly decreased mRNA levels for *Srebp1c* and *Tgfb*, but not for *Serpine1* in the reverse experiment, whereas metformin decreased mRNA levels for *Serpine1*, but not for *Srebp1c* and *Tgfb* in the preventive experiment. These findings suggest that metformin may ameliorate hepatic steatosis and fibrosis both via SREBP-1c- and TGF-b-dependent pathways and via a PAI-1-mediated pathway. Anyway, further time-course experiments should be required for confirm direct effects of metformin on the genes involved in steatosis and fibrosis to address these hypothesis.

In conclusion, metformin may prevent and reverse steatosis and inflammation in a nondiabetic mouse model of steatohepatitis possibly by downregulating pleiotropically lipogenic and fibrogenic genes such as *Serpine1* without affecting insulin resistance. The present findings suggest the therapeutic potential of metformin in human NASH even in patients without diabetes. Large scale clinical trial should be required to test the hypothesis that metformin reverses pathology of NASH in non-diabetic patients.
